# Advanced age, altered level of consciousness and a new diagnosis of diabetes are independently associated with hypernatreamia in hyperglycaemic crisis

**DOI:** 10.1186/1472-6823-11-8

**Published:** 2011-04-18

**Authors:** Chukwuma O Ekpebegh, Benjamin Longo-Mbenza, Augustin Nge-Okwe, Anthonia O Ogbera, Nomawethu T Tonjeni

**Affiliations:** 1 Department of Internal Medicine, Faculty of Health Sciences, Walter Sisulu University/Nelson Mandela Academic Hospital, Mthatha, Eastern Cape Province, South Africa; 2 Biostatistics Unit, Lomo Medical Center and Heart of Africa Centre of Cardiology, Kinshasa, Democratic Republic of Congo; 3 Department of Internal Medicine, Lagos State University Teaching Hospital, Ikeja, Lagos State, Nigeria

**Keywords:** Hypernatreamia, Hyperglyceamic crisis, prevalence, determinants, South Africa

## Abstract

**Background:**

There is limited literature on hypernatreamia in the setting of hyperglycaemic crisis. This is despite the fact that the presence of hypernatreamia may impact on the classification of hyperglycaemic crisis and its management particularly with regards to the nature of fluid therapy. We determined the prevalence of hypernatreamia and its associated factors at presentation for hyperglycaemic crisis.

**Methods:**

This was a retrospective review of data for hyperglycaemic crisis admissions in Nelson Mandela Academic Hospital, Mthatha, South Africa. The prevalence of hypernatreamia (uncorrected Serum Sodium at presentation >145 mmol/L) was determined. Hyperosmolality was defined by calculated effective osmolality >320 mosmols/Kg. Multivariate logistic regression was undertaken using variables that were statistically significant in univariate analysis to ascertain those that were independently associated (Odds Ratio (OR) with 95% Confidence Interval (CI)) with hypernatreamia.

**Results:**

The prevalence of hypernatreamia in our admissions for hyperglycaemic crisis was 11.7% (n = 32/273 including 171 females and 102 males). All admissions with hypernatreamia met the criteria for hyperosmolality. Age ≥ 60 years (OR = 3.9 95% CI 1.3-12.3; P = 0.018), Altered level of consciousness (OR = 8.8 95% CI 2.3-32.8; P < 0.001) and a new diagnosis of diabetes (OR = 3.7 95%CI 1.2-11.5; P = 0.025) were independently associated with hypernatreamia.

**Conclusion:**

The prevalence rate of hypernatreamia in hyperglycaemic admissions was high with all hypernatreamic admissions meeting the criteria for hyperosmolality. Advanced age, altered conscious level and a new diagnosis of diabetes were independently associated with hypernatreamia.

## Background

Serum sodium measurement is useful in the management of hyperglycaemic crisis as it enables the determination of serum osmolality and anion gap [1]. Hyponatreamia, albeit, a pseudo phenomenon is the predominant serum sodium abnormality reported in hyperglycaemic emergencies [2,3]. While there is considerable literature on hypernatreamia in general [4-7], data on hypernatreamia occurring in the context of hyperglycaemic crisis is limited. Hypernatreamia can have varied aetiology including diabetes insipidus, mineralocorticoid excess, infusions of hypertonic saline and sodium bicarbonate [5]. However, a raised serum sodium level at presentation of hyperglycaemic crisis usually reflects marked dehydration due to excessive free water loss from osmotic diuresis [8]. Measures such as vigorous fluid replacement, use of hypotonic fluids and prophylactic anticoagulation may be recommended in the setting of hypernatreamia complicating hyperglycaemic crisis. We assessed the prevalence and factors associated with hypernatreamia in a cohort of patients admitted with hyperglycaemic crisis at the Nelson Mandela Academic Hospital (NMAH). The NMAH is a tertiary public health facility situated in the Eastern Cape Province of South Africa and serves a predominantly indigent rural population of about 1.7 million people. The NMAH receives referrals from primary and secondary health care facilities in its area of drainage.

## Methods

This is a retrospective review of hospital records of admissions for hyperglycaemic crisis during the two year periods of 2008 and 2009. Readmissions of the same patient were included as the levels of serum sodium at presentation may vary in different admissions. The prevalence of hypernatreamia at presentation (as a percentage of total admissions for hyperglycaemic crisis) and its associated factors were determined. Ethical approval was obtained from the Walter Sisulu University.

### Definitions

Hypernatreamia was defined as uncorrected serum sodium at presentation above 145 mmol/L, hypotension as systolic blood pressure (SBP) < 90 mm/Hg, hyperosmolality as calculated effective osmolality [9] >320 mosmols/Kg, hyperchloreamia as serum chloride >104 mmol/L, elevated serum urea as serum urea >7 mmol/L, leukocytosis as white blood count (WBC) >10 × 10^6^/mm^3 ^and thrombocytopenia as platelet count <150 × 10^6^/mm^3^. Types of hyperglycaemic crisis as (a) non-hyperosmolar ketoacidosis: blood glucose >13.9 mmol/L with serum bicarbonate <18 mmol/L and calculated effective osmolality ≤ 320 mosmols/Kg, (b) hyperosmolar ketoacidosis: blood glucose >13.9 mmol/L with serum bicarbonate <18 mmol/L and calculated effective osmolality >320 mosmols/Kg, (c) hyperosmolar non-ketotic state: blood glucose >33.3 mmol/L with serum bicarbonate ≥ 18 mmol/L and calculated effective osmolality >320 mosmols/Kg, and (d) hyperglycaemia: blood glucose >13.9 mmol/L with serum bicarbonate ≥ 18 mmol/L and calculated effective osmolality ≤ 320 mosmols/Kg were as previously defined [10]. Hyperglycaemia without ketoacidosis or hyperosmolality was considered to be in crisis where treatment with intravenous fluids and insulin infusion were considered necessary by the attending medical staff.

### Statistical analysis

Continuous and categorical variables were expressed as mean ± SD and percentages (%) respectively. Mean of continuous variables were compared with students t test while categorical variables were compared with Chi-square test. The prevalence of hypernatreamia was determined and factors associated with hypernatreamia were assessed using univariate and multivariate analysis. The multivariate Odds ratios (OR) and corresponding 95% confidence intervals (95%CI) were obtained in logistic regression model. A P-value < 0.05 was taken as statistically significant. All data analysis was done with statistical package for social sciences (SPSS) software version 18 for windows (SPSS Inc, Chicago, Il, USA).

## Results

There were 273 admissions with serum sodium results. Four of these 273 admissions who only had blood glucose readings as 'high' on glucometer testing and no documented laboratory blood glucose values in their case records were excluded from any analysis involving type of hyperglycaemic crisis as the serum osmolality could not be calculated to allow for categorization into one of the 4 types of hyperglycaemic crisis. These 4 admissions were also excluded from any analysis involving corrected serum sodium. Ten individuals were admitted more than once with a range of 2-7 admissions. One hundred and seventy one (171) and 102 admissions were female and male related respectively. The proportions of admissions for the various types of hyperglycaemic crisis were: DKA (45%, n = 121/269); 36.1% (n = 97/269) for non-hypersomolar DKA and 8.9% (n = 24/269) for hyperosmolar DKA. Hyperosmolar non-ketotic state and hyperglycaemia accounted for 10.8% (n = 29/269) and 44.2% (n = 119/269) of all admissions respectively. The prevalence of uncorrected hypernatreamia was 11.7% (n = 32/273) while the prevalence was 30.1% when corrected for blood glucose concentration using the formula: corrected Serum Sodium in mmol/L = Serum Sodium in mmol/L + 1.6 (Plasma glucose in mmol/L - 5.5 mmol/L)/5.5. Table 1 shows that the majority of hypernatreamic admissions, had serum sodium levels in the range of 146-155 mmol/L with few admissions associated with a presenting uncorrected or corrected serum sodium above 165 mmol/L. Table 2 shows similar gender distribution among hypernatreamic and non-hypernatreamic admissions. Both groups had comparable mean ages but the proportion of admissions associated with age ≥ 60 years was more in the hypernatreamic than non-hypernatreamic admissions. The mean heart rate (111.6 ± 16.7 versus 100.7 ± 21 beats per minute, P = 0.03) was higher in the hypernatreamic than non-hypernatreamic admissions. Systolic blood pressure (111 ± 29.1 versus 128.9 ± 27.9 mm/Hg, P = 0.04) and diastolic blood pressure (65.1 ± 22.7 versus 78.8 ± 19.2 mm/Hg, P = 0.002) were both lower in the hypernatreamic than non-hypernatreamic admissions. Table 2 also shows that hypotension, altered level of consciousness and a new diagnosis of diabetes mellitus were all significantly associated with hypernatreamia in univariate analysis. Table 3 shows admissions with hypernatremia at presentation to be associated with hyperosmolality, hyperchloreamia, leukocytosis, thrombocytopenia and elevated serum urea levels.

**Table 1:** Proportions of admissions with various degrees of hypernatreamia based on uncorrected and corrected serum sodium levels.

	Hypernatreamia based on uncorrected Serum sodium N = 273	Hypernatreamia based on corrected serum sodium N = 269
146-155 mmol/L	6.2% (n=17/273)	16% (n=43/269)
156-165 mmol/L	4.8% (n=13/273)	10.8% (n=29/269)
>165 mmol/L	0.7% (n=2/273)	3.3% (n=9/269)

**Table 2:** Comparison of demographic and clinical variables in hypernatreamic and non-hypernatreamic admissions based on uncorrected serum sodium >145 mmol/L

	Hypernatreamia	Non-Hypernatreamia	P value
Age (years)	51.7 ± 21.4 (n = 32/32)	50.2 ± 19.6 (n = 241/241)	0.7
Females (%)	68.8 (n = 22/32)	61.8 (n = 149/241)	0.4
Age ≥ 60 years (%)	53.1 (n = 17/32)	34.4 (n = 83/241)	0.04
Hypotension (%)	30.4 (n = 7/23)	6.1 (n = 11/181)	<0.00001
Altered level of consciousness (%)	88.9 (n = 24/27)	32.5 (n = 54/166)	<0.00001
New diagnosis of diabetes (%)	46.9 (n = 15/32)	25.7 (n = 62/241)	0.01

**Table 3:** Comparison of biochemical variables in hypernatreamic and non-hypernatreamic admissions based on uncorrected serum sodium >145 mmol/L

	Hypernatreamia	Non-Hypernatreamia	P value
Hyperosmolality (%)	96.9 (n = 31/32)	9.3 (n = 22/237)	<0.0001
Hyperchloreamia (%)	93.8 (n = 30/32)	13 (n = 31/239)	<0.0001
Leukocytosis (%)	83.9 (n = 26/31)	60.7 (n = 142/234)	0.01
Thrombocytopenia (%)	32.3 (n = 10/31)	9.4 (n = 22/234)	<0.00001
Elevated Serum Urea (%)	27.6 (n = 8/29)	54.9 (n = 130/237)	0.006
HbA1C >7% (%)	94.1 (n = 16/17)	77.4 (n = 123/159)	0.107
Blood glucose (mmol/L)	33.9 ± 18.7 (n = 32)	32.4 ± 15.7 (n = 237)	0.6

The independent effects of factors that were significantly associated with hypernatreamia were investigated in multivariate analysis using stepwise forward Wald logistic regression. There was colinearity between elevated serum urea level and hyperchloreamia in the model and so hyperchloreamia was excluded from the logistic regression analysis. The multivariate OR of hypernatreamia was then calculated with its 95% CI. The multivariate analysis identified age ≥ 60 years, altered level of consciousness and a new diagnosis of diabetes as significant and independent determinants of the presence of hypernatreamia at admission. Table 4 shows that the risk of hypernatreamia was increased 4 times respectively for admissions associated with age ≥ 60 years and a new diagnosis of diabetes. The risk for hypernatreamia was 10 fold higher with altered level of consciousness.

**Table 4:** Independent and Significant determinants of Hypernatreamia [based on uncorrected serum sodium >145 mmol/L] using logistic regression model

Independent variables	Beta Coefficient	Standard Error	Wald Chi-square	Odd Ratio (95% Confidence Interval)	P value
Age groups: ≥60 years versus <60 years	1.371	0.581	5.570	3.9 (1.3-12.3)	0.018
Altered level of consciousness: Yes versus No	2.172	0.673	10.404	8.8 (2.3-32.8)	<0.001
New diagnosis of Diabetes: Yes versus No	1.301	0.581	5.015	3.7 (1.2-11.5)	0.025
Constant	-4.489	0.750	35.784		<0.0001

Adjusted for systolic blood pressure, white cell count, platelet count and serum urea levels.

## Discussion

The major findings of this study are a hypernatreamia prevalence rate of 11.7% based on uncorrected serum sodium >145 mmol/L and 30.1% based on a serum sodium of >145 mmol/L corrected for the ambient blood glucose concentration and the independent association of hypernatreamia with advanced age, altered level of consciousness and a new diagnosis of diabetes. The prevalence rate of 11.7% that we observed for uncorrected hypernatreamia is much higher than the 1.2% reported in another study [2] with similar definition of hypernatreamia (uncorrected serum sodium >145 mmol/L). Our admissions consisted of ketoacidosis, hyperosmolar non-ketotic state and hyperglycaemia with hypernatreamia rates of 17.4%, 37.9% and 0% respectively. Further analysis of our ketoacidosis patients revealed a hypernatreamia rate of 83.3% for hyperosmolar ketoacidosis and 1.2% for non-hyperosmolar ketoacidosis. Perhaps, the study [2] with a hypernatreamia rate of 1.2% was predominantly patients with non-hyperosmolar ketoacidosis. Although hyperosmolar DKA is increasingly being reported in children [11,12] and adults [13,14], their hypernatreamia rates were not documented. The proportions of hyperglycaemic crisis admissions presenting with hyperosmolar DKA was 15.1% (n = 8/53) in one study [13], and 45% (n = 288/613) in another study [14].

As uncorrected serum sodium >145 mmol/L was found in none of 119 hyperglycaemic and only 1 of 97 non-hyperosmolar ketoacidosis admissions, the specificity of uncorrected serum sodium >145 mmol/L for the diagnosis of hyperosmolality was 99.5% based on calculated effective osmolality >320 mosmols/kg. The sensitivity of uncorrected serum sodium >145 mmol/L for the diagnosis of hyperosmolality was 62.6% as 20 of 24 admissions for hyperosmolar ketoacidosis and 11 of 29 admissions for hyperosmolar non-ketotic state had uncorrected serum sodium >145 mmol/L. Thus while almost all admissions with presenting uncorrected serum sodium >145 mmol/L were hyperosmolar, not all hyperosmolar admissions had initial uncorrected serum sodium level >145 mmol/L. This suggests that in our setting, hyperglycaemic patients with uncorrected serum sodium levels >145 mmol/L should be managed as hyperosmolar states.

A study [15] that was conducted on patients admitted to an intensive care unit reported the mechanisms for hypernatreamia to include salt overload and fluid depletion with the use of sodium bicarbonate, mannitol, impaired urinary concentration and sepsis as the independent determinants of hypernatreamia. Unlike our patients who were already hypernatreamic at presentation, the patients in this study [15] developed hypernatreamia in the course of hospitalization. Sodium bicarbonate, mannitol or hypertonic saline could not have been contributory to the hypernatreamia in our patients as there was no prior administration of these agents at their referring hospitals before presentation to us. In another study [16], 50% of patients who developed hypernatreamia during hospitalization and 89% of patients presenting with hypernatreamia had urinary concentration defects primarily associated with diuretic therapy or solute diuresis. Although, our patients with hyperglycaemic crisis will expectedly have glycosuria induced diuresis, hypernatremia was mainly a problem in those who were elderly, had altered sensorium or were newly diagnosed with diabetes.

The independent association of age ≥ 60 years with hypernatreamia may be partly explained by an increased threshold for thirst and vasopressin deficiency that is associated with ageing [17,18]. Therefore, patients with advanced age may become hypernatreamic due to inadequate compensatory increased oral fluid intake and renal fluid retention in the face of glycosuria induced osmotic diuresis. Altered level of consciousness, regardless of aetiology may result in hypernatreamic dehydration because the patient is unable to replenish renal fluids loss orally due to impairment of the mental state. As our study was not only retrospective but cross-sectional in design, we can only state that impaired mental state was associated with hypernatreamia. The study design does not permit for the exploration of a causal relationship between hypernatreamia and altered level of consciousness. A report [19] which found all patients with hyperosmolar non-ketoacidotic state to have altered level of consciousness did not indicate the serum sodium levels or any association of hypernatreamia to coma. It is interesting that a new diagnosis of diabetes was significantly independently associated with hypernatreamia. Although admission blood glucose levels was non-statistically higher in the newly diagnosed than known diabetic patients (35.35 ± 19.0 mmol/L versus 31.5 ± 14.6 mmol/L, P = 0.079), the proportion of admissions with HbA1c level above 10% was more in the newly diagnosed than known diabetic patients (87.7% versus 74.8%, P = 0.049). This suggests that the majority of patients with hyperglycaemic crisis as the first manifestation of diabetes had more severe chronic hyperglycaemia than previously diagnosed diabetic patients. Perhaps, these newly diagnosed diabetic patients consequently had more prolonged osmotic diuresis with hypernatreamic dehydration.

The findings from this study suggest that particular attention should be given to serum sodium levels in admissions for hyperglycaemic crisis associated with advanced age, unconsciousness at presentation and a new diagnosis of diabetes. These patients will require more attention to fluids therapy and may require prophylactic anticoagulation as all hypernatreamic admissions met the criteria for hyperosmolality.

### Limitations of study

The limitations of this study include its retrospective design and determination of serum osmolality by calculation rather than laboratory measurement. We may have underestimated serum osmolality where osmotically active substances other than glucose and sodium are present in the serum as we calculated rather than measured the serum osmolality. Another limitation is that results of urine osmolality, urine specific gravity and urine electrolytes concentrations were not provided as these are not routinely done in our practice.

## Conclusions

In this retrospective review of hospital records for hyperglycaemic crisis, we observed prevalence rate for hypernatreamia of 11.7% based on uncorrected serum sodium concentration. All hypernatreamic admissions met the criteria for hyperosmolality. Advanced age, altered conscious level and a new diagnosis of diabetes were independently associated with hypernatreamia.

## Competing interests

The authors declare that they have no competing interests.

## Authors' contributions

COE conceived the study, participated in its design, collation of data and drafting of the manuscript. BLM participated in the study design, statistical analysis and interpretation of data and revising the manuscript for important intellectual content. AN participated in the study design, statistical analysis and interpretation of data. AO participated in the study design and drafting of the manuscript. NTT participated in the study design and revision of the manuscript critically for important intellectual content. All authors read and approved the manuscript.

## Pre-publication history

The pre-publication history for this paper can be accessed here:

http://www.biomedcentral.com/1472-6823/11/8/prepub
